# Activation of Person Knowledge in Medial Prefrontal Cortex during the Encoding of New Lifelike Events

**DOI:** 10.1093/cercor/bhab027

**Published:** 2021-04-19

**Authors:** Petar P Raykov, James L Keidel, Jane Oakhill, Chris M Bird

**Affiliations:** School of Psychology, University of Sussex, Falmer BN1 9QH, UK

**Keywords:** fMRI, memory, medial prefrontal cortex, prior knowledge, schema

## Abstract

Our knowledge about people can help us predict how they will behave in particular situations and interpret their actions. In this study, we investigated the cognitive and neural effects of person knowledge on the encoding and retrieval of novel life-like events. Healthy human participants learnt about two characters over a week by watching 6 episodes of one of two situation comedies, which were both centered on a young couple. In the scanner, they watched and then silently recalled 20 new scenes from both shows that were all set in unfamiliar locations: 10 from their trained show and 10 from the untrained show. After scanning, participants’ recognition memory was better for scenes from the trained show. The functional magnetic resonance imaging (fMRI) patterns of brain activity when watching the videos were reinstated during recall, but this effect was not modulated by training. However, person knowledge boosted the similarity in fMRI patterns of activity in the medial prefrontal cortex (MPFC) when watching the new events involving familiar characters. Our findings identify a role for the MPFC in the representation of schematic person knowledge during the encoding of novel, lifelike events.

## Introduction

We often rely on our prior knowledge to understand the world around us. Knowing a friend’s personality, temperament, and their likes and dislikes can help us understand and predict their behavior in a given situation. Such “person knowledge” can be considered as a type of schematic knowledge (Baldwin 1992; Ramon and Gobbini 2018). Schemas are memory structures learned over multiple episodes that highlight features commonly occurring across events (Ghosh and Gilboa 2014; Gilboa and Marlatte 2017). Here, we investigated the cognitive and neural effects of person knowledge on the encoding and retrieval of novel naturalistic events.

Many studies have demonstrated that person knowledge has a profound influence on how we process information in a wide range of situations. For example, people are better at identifying and remembering photos of familiar versus unfamiliar individuals (Klatzky and Forrest 1984; Trinkler et al. 2009; Bird et al. 2011; Liu et al. 2016; Ramon and Gobbini 2018; Raykov et al. 2020). In a more “real-world” setting, Vazire and Mehl (2008) measured the amount of time individuals spent on different daily activities and found that people are able to estimate these times for a close friend as well as they are able to estimate the times for themselves (see also Tamir and Thornton 2018).

Person knowledge can also affect what we remember from a specific event. Cohen (1981) presented participants with a video clip showing the daily activities of a woman. Before watching the clip, some participants were informed that the woman is a librarian, whereas another group were told she was a waitress. Participants showed better memory for features of the video that were consistent with the information that they were provided with beforehand. Interestingly, this memory benefit was not observed if the knowledge was provided after having seen the clip. This suggests that person knowledge acquired prior to watching the clips biased how participants encoded the information (see also Dooling and Christiaansen 1977; Baldwin 1992).

Schema-based memory processing has been associated with a number of brain regions, with the medial prefrontal cortex (MPFC) being the most consistently implicated (e.g., van Kesteren et al. 2012; Preston and Eichenbaum 2013; Gilboa and Marlatte 2017; Robin and Moscovitch 2017). Considering person knowledge specifically, tasks involving simply viewing, or making semantic judgments about, known individuals (for whom schematic knowledge is available), frequently engage both the MPFC and posterior midline regions (e.g., Leveroni et al. 2000; Gobbini et al. 2004; Elfgren et al. 2006; Horner et al. 2015; Liu et al. 2016; di Oleggio Castello et al. 2017; Ramon and Gobbini 2018; Raykov et al. 2020). Recently, Raykov et al. (2020) familiarized participants with a television situation comedy and found that viewing photos of the characters from the familiar show activated the MPFC and posterior midline cortex (PMC) when compared with viewing characters from a similar, but unfamiliar, show. Taken together, these studies identify the MPFC and PMC as key regions involved with the processing of person knowledge as well as with memory schemas more generally. However, to date, tasks investigating the activation of schema-related information have mainly used static images or verbal labels rather than more extended naturalistic events.

An exception to this is a recent study that investigated “scripts,” which are representations of stereotyped everyday situations that comprise a consistent set of characters, props, and sequences of actions (e.g., eating at a restaurant) (Baldassano et al. 2018). Participants watched or listened to short event sequences based in a restaurant or airport—both situations involving well-established activities such as ordering food or going through security. The authors found that fMRI patterns of activity, notably in the MPFC and PMC, were similar when people activated the same general script, even if the surface features of the events (the specific storylines and characters) were quite different from each other.

In this study, we investigate the influence of person knowledge on the processing of complex, lifelike events. We focus on how the MPFC and PMC are recruited when watching familiar people in novel situations. Therefore, in contrast to the study of script processing by Baldassano and colleagues, participants viewed events that differed in situations depicted, but involved the same pairs of characters (young male and female couples). All events were taken from two television situation comedies, which are centered on a main couple. Prior to fMRI scanning, participants watched 6 “training” episodes from one of the two shows to familiarize themselves with the characters from the show. Inside the scanner, participants watched and retrieved previously unseen short clips taken from the trained and the untrained show. Each clip depicted a situation in a unique location (e.g., a museum, a park) involving the main characters of the show.

Through watching the training episodes, participants not only acquire knowledge about the two main characters but also other aspects of the show more generally. Therefore, viewing the novel scenes from the shows in the scanner might be expected to trigger the retrieval of this wider body of knowledge. However, our task was constructed in order to strongly bias the retrieval of person knowledge versus more general information about the shows. First, the shows are matched at the most general level: both are romantic situation comedies aired in the United States of America during the 1990s and which focus on a relatively young, childless, couple. Therefore, this general knowledge should be activated by viewing both shows. Second, in order to attempt to minimize differences between the shows in terms of cinematic features such as the lighting and the editing, all the clips showed single scenes presented at the same volume without color. Third, the only familiar elements in any of the scenes shown in the scanner were the main couples from the shows—all other characters and the locations were unfamiliar. Finally, both shows revolve almost exclusively around the main characters: almost every scene from the training episodes features one of the two main characters and they are involved in all of the storylines and directly associated with all of the other characters in the show. Therefore, any overarching “show level” knowledge is intrinsically linked to the shows’ main characters.

To seek evidence for the activation of person-specific schemas, we investigated whether different events that involve the characters from the trained show are more similar to each other than the videos taken from the untrained show. To compare our study with more conventional designs that have compared photos of known versus unknown individuals, we compared overall activity between the trained and untrained clips at both encoding and retrieval. Our final analyses investigated whether reinstatement of patterns of brain activity between encoding and recall of the clips was modulated by training. We predicted that participants would have richer representations of the events involving known individuals, resulting in greater reinstatement effects for the video clips from the trained show.

## Materials and Methods

### Participants

The participants were the same as those who took part in the study by Raykov et al. (2020). The study recruited 30 right-handed native English speakers (15 females) aged 18–29 years (21.71 ± 3.08). One participant did not complete the experiment due to a technical issue with the scanner, one participant was excluded because their data were corrupted due to a technical issue, and one participant was excluded from the main analyses due to poor memory performance for the clips presented in the scanner. Twenty-seven participants were included in the fMRI and behavioral analyses. Informed consent was obtained from all participants and they were each paid £40.

**Figure 1:**
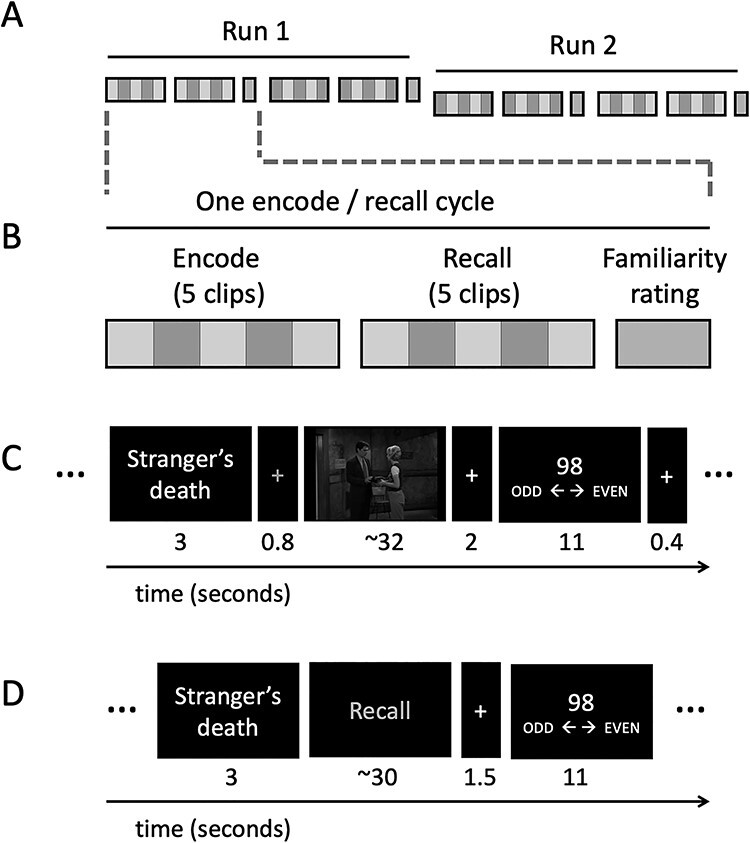
Study design. In the week before the experiment (not shown in the figure), participants were familiarized with one out of two shows. Participants then viewed and recalled silently clips from the trained and untrained show in the scanner. (*A*) There were 20 clips in total separated into 2 runs. In each run, participants encoded and recalled 10 clips (5 trained and 5 untrained) across 2 encode/recall cycles. (*B*) Participants watched 5 clips (including both trained and untrained clips) and then recalled them in a different order. After each encode/recall cycle participants rated their familiarity with the characters in the videos. (*C*) Shows the timings for encoding phases. (*D*) Shows the timings for the recall phases. After both encode and recall trials, participants made an odd-even judgment as an active baseline task.

### Stimuli

The stimuli used for the study comprised (1) 6 full episodes of two situation comedy shows (“Mad About You” – MAY and “Dharma and Greg” – DaG) that were used for training, (2) 64 color pictures from the two shows, (3) 20 short clips taken from episodes of the two shows not seen during training, and (4) 100 three-alternative forced-choice questions to assess recognition memory for details from the short clips (see Supplementary Fig. 1 for example, and Supplementary Materials for list of all questions). Full details about the pictures task, included fMRI findings, are described in Raykov et al. (2020) and are not reported here except in the Supplementary Materials.

Both shows focus on the lives of a couple in their 30s (Jamie and Paul in MAY, Dharma and Greg in DaG) and are set in the 1990s in the USA. The shows were chosen to be unfamiliar to our participants. We also excluded potential participants who were familiar with other shows/films where the same actors played the main characters. Each of the training episodes from both shows was approximately 21 min in length and did not include commercial breaks. The training episodes were not taken from consecutive episodes and focused on different events from the main characters’ lives (see Supplementary Materials for list of episodes). In each episode, there was a main overarching theme (e.g., having friends over for dinner; finding new friends) that provided a general setting for the string of events happening in the episode. Most of the scenes in the training episodes involved dialog between the main characters in their apartment. All storylines directly related to the main couple in the show even if they involved additional characters. Indeed, 98% of all screen-time in the training episodes showed scenes that included at least one of the main characters.

Twenty short clips were used in the scanning session (see Supplementary Materials for the full list of clips used). All clips used in the scanning session depicted self-contained short situations that were unrelated to each other. The clips were taken from previously unseen episodes and all took place in single unfamiliar locations (e.g., the museum). The duration of the 10 clips from MAY (32.7 ± 6.73) were on average the same duration as clips from DaG (33.4 ± 7.87) (*P* = 0.833). The audio for the clips was scaled to the same mean decibel intensity with Praat (version 6.0.15) (Boersma 2001). To control for potential differences in the color of the clips, they were all converted to black and white. Multiple-choice questions were created to test participants’ memory for the short clips (see Supplementary Materials for all the questions used).

### Procedure

Participants were assigned to either the MAY or DaG training condition in a counter-balanced order. Participants were provided with 6 episodes from one of the shows and were asked to watch them in their own time over the course of the week, rather than in one sitting. To ensure that all of the 6 training episodes had been watched, participants were asked to describe them, and their memory was probed for specific details (e.g., what was said, the intentions of the characters, the location). This informal screening procedure lasted for approximated 45 min and was carried out before booking the scanning session. On the basis of their responses, one participant was asked to re-watch one of the training episodes.

Participants carried out 4 functional runs within the scanner. The first and last of the 4 runs involved the picture task reported previously. The middle 2 runs are the focus of this paper. Each run was approximately 16 min long. In each run, participants encoded and silently recalled 10 clips in total (5 from the trained show and 5 from the untrained show) (see Fig. 1*A*). In order to reduce memory demands, participants encoded and recalled the clips in sets of 5 (e.g., encode 5 videos and then recall these 5 clips) (see Fig. 1*B*). Within each set there were both trained and untrained clips presented in random order. Before each video, participants were presented with a title associated with the video (e.g., A Stranger’s Death) for 3 s. Participants were instructed that the title would act as a memory cue later on and were asked to pay attention to it. The title was followed by a red cross (see gray cross in Fig. 1*C*) that allowed us to lock the onset of the clips to the start of a scan (functional brain volume acquisition). Each clip was followed by a 2 s white fixation cross after which participants were asked to make an odd/even number judgment for 11 s, which served as an active baseline task (Stark and Squire 2001; Raykov et al. 2020). A white fixation cross lasting for 400 ms was presented before the onset of the next title. See Figure 1*C* for the timings of the encoding phase.

After encoding the 5 videos, participants silently recalled them in a random order. Participants were cued with the video’s title (e.g. A Stranger’s Death) for 3 s and then instructed to “Recall.” The recall cue stayed on the screen until participants made a response to indicate that they had finished recalling or until 30 s had elapsed. Each recall event was followed by a white fixation cross for 1.5 s and then the odd/even number task for 11 s. See Figure 1*D* for the timings of the recall phase.

After completing each of the four encode/recall cycles, participants were presented with visual analog scales (VAS) on which they rated their familiarity with each of the 4 characters. Each scale was presented for 6 s and participants rated from 0 to 100 how familiar they felt with each character.

The scanning session was immediately followed by a testing session comprising three parts. First, for each video, a short free recall test was administered to establish how effective the titles cues were at eliciting recall of the cued clip. This was followed by a rating task and lastly, a recognition test for details from the clips.

In the free recall test, the title of each clip was presented on a computer screen and participants were asked to type out a brief summary of the content of the clip. Answers were scored as 0 if the response was clearly incorrect, left blank, “Do not remember” (or similar), or correctly identified which show was involved but gave no further details. Answers that correctly identified a feature that was unique to the specific clip were scored as 1 (e.g., “Paul and Jamie meet and discuss their families and first dates”).

In the rating task, participants were shown each title and asked to rate on a 0–100 VAS how engaging they found the clips and how vividly they could recall them.

In the recognition test, participants completed a three-alternative forced-choice memory test concerning details about the clips they viewed in the scanner (five questions per clip). The sets of 5 questions were presented in a pseudo random order, so that there were no more than 3 questions in a row for the same show. The proportion correct responses were calculated for each show, with chance level being 0.33. We then averaged these proportions separately for each participant and each condition and compared memory performance for the trained and the untrained clips. The responses to the multiple-choice questions were our main measure of memory for the clips.

### MRI Acquisition

T2^*^-weighted fMRI images were acquired on a 3 T Siemens Prisma scanner using a 32-channel head-coil. To minimize movement, soft cushions were inserted into the head coil. We used an fMRI sequence initially developed as part of the Human Connectome Project (Van Essen et al. 2012). A gradient-echo EPI sequence with multiband acceleration factor of 8 with the following parameters (TR = 0.8 s; TE = 33.1 ms; 52° flip angle; FOV = 208 x 180 mm; 72 sliced with sliced thickness of 2 mm and isotropic 2 mm voxels). The same parameters were used to acquire two SpinEcho Fieldmap runs with reversed phase-encode blips in both anterior to posterior and posterior to anterior directions. These pairs of images were used to estimate the distortion field map using a method similar to Andersson (2003) as implemented in FSL. A T1-weighted high-resolution structural image was acquired with 3D MPRAGE sequence (TR = 2.4 s; TE = 2.14 s; 8° flip angle; FOV = 224 x 224 mm and 0.8 mm isotropic voxels).

### Image Preprocessing

We used SPM 12 (Wellcome Department of Imaging Neuroscience, London, UK) to preprocess all the images except the field maps. Images from both sessions were spatially realigned to the mean functional image to account for any motion. Command-line functions from FSL (Smith et al. 2004) were used to estimate and apply field maps to the motion corrected data in order to correct for image distortions (Andersson et al. 2001). The high-resolution structural image was coregistered to the mean functional image and was segmented into gray, white matter, and cerebrospinal fluid using tissue probability maps. The segmented images were used to estimate deformation fields, which were applied to the functional images in order to transform them to MNI space. A 6-mm FWHM smoothing kernel was applied to the functional images. Unsmoothed normalized images were used for the region of interest (ROI) analyses.

### Data Analysis

Data were analyzed with SPM 12, the CosMoMVPA toolbox (Oosterhof et al. 2016), and custom scripts in MATLAB (Version 2017b, The MathWorks, Inc.). All analyses were conducted on MNI normalized images. The RobustWLS toolbox in SPM 12 was used to estimate the first level models (Diedrichsen and Shadmehr 2005). This method provides a “soft” exclusion of bad volumes by down-weighting volumes with high variance estimates. We used the Bspmview toolbox (www.bobspunt.com/bspmview) to visualize and describe our data. The toolbox implements MNI coordinates from the Anatomical Automatic Labelling 2 toolbox for SPM 12.

### ROI Definition and Analyses

We focused our analyses on the MPFC and PMC (see Introduction). Both ROIs are taken from the dorsal default mode network identified by Shirer et al. (2012) (see Fig. 3*A*). We wished to compare ROIs of approximately equal size, so that any differences between the regions could not be due to a difference in the number of voxels entered into the analysis. Therefore, we selected voxels within the center of the larger MPFC region (all voxels within a 16 mm sphere aligned to the center of mass of the original ROI; see Supplementary Fig. 7 for results from original ROI). Univariate analyses (see below) are based on the average response of all (unsmoothed) voxels within each ROI. Multivariate analyses (see below) are based on the pattern of activity across all (unsmoothed) voxels within each ROI.

### Whole-Brain Analyses

We also carried out whole-brain random-effects analyses across participants, which were performed on the normalized, smoothed images. For the univariate analyses (see below), first-level contrast images from each participant were evaluated in second-level (group) analyses with one-sample *t*-tests. For the multivariate analyses (see below), a spherical searchlight with radius 4 voxels (mean searchlight size = 235 voxels) was used. Unless otherwise stated, results were thresholded in SPM using cluster-level family-wise error correction (*P* < 0.05), with a cluster-defining threshold of *P* < 0.001.

### General Linear Models

For the univariate analyses, the general linear models (GLM) included a single task regressor for each of the 4 conditions (Train Encode, Train Recall, Untrain Encode, Untrain Recall). The title cues were modeled with a single regressor of no interest and an additional regressor for the familiarity rating was included. The regressors included the onset and whole duration of trials (encode or recall). For the recall trials, the durations are determined by when the participant pressed the button to terminate the trial. We report the comparisons of brain activity between the trained and untrained videos at both encoding and recall.

We also ran an exploratory analysis comparing the trained and untrained video clips when only modeling the onset of the clip, which is reported in Supplementary Figure 2. This was in order to make a qualitative comparison between the results of the fMRI task for pictures of the main characters reported in Raykov et al. (2020) and the responses to the video onsets.

For the multivariate representational similarity analyses (see below), we ran separate first-level GLMs where we modeled the full duration of each encode (20) and recall (20) event with a separate regressor. Given the slow-event related design, we modeled each trial as a separate regressor in a single first-level model as in the least-squares all (LSA) method described in Mumford et al. (2012). This allowed us to examine video specific patterns and whether these patterns were reinstated during recall.

### Representational Similarity Analysis

The GLM estimated single trial t-maps were subjected to representational similarity analyses. We investigated the similarity between spatial patterns of BOLD activity during encoding and recall of the videos using different representational similarity analyses (RSAs) (Kriegeskorte et al. 2008). We focus on the results from our two ROIs but also report whole-brain searchlight analyses for completeness. For all analyses, we computed the multivoxel spatial pattern similarity across pairs of trials using correlation. The Pearson’s correlation values were then Fisher transformed and weighted according to a contrast matrix (see Fig. 3). Different contrast matrixes were used for the different analyses described below, although in all cases, the matrix summed to zero.

To investigate effects of training during encoding, we compared the pattern of brain activity across all video clips from the trained show to the pattern of brain activity across clips from the untrained show (see Fig. 3*B*). We tested whether the patterns of activity associated with clips from the trained show are more similar to each other than to those associated with clips from the untrained show. The autocorrelation diagonal which corresponds to the correlations between identical clips is not included (since all values are 1). It is important to note that each clip involved a unique topic of conversation and was set in a unique and untrained location; therefore, any effects are likely to be attributable to the characters present. Note, in Supplementary Materials, we also report an exploratory timepoint-by-timepoint analysis showing the comparison of similarity among trained and untrained clips during encoding for each successive TR (see Supplementary Figs 3–6).

Two posthoc analyses were conducted to further investigate the relationship between pattern similarity during encoding and recognition memory performance outside of the scanner. We first analyzed the simple correlation between the boost in accuracy to the trained versus untrained memory clips and the MPFC trained versus untrained RSA similarity effect. Second, to more formally examine whether MPFC trained versus untrained pattern similarity boosted memory accuracy for the trained clips, we fitted a random intercept logistic mixed effects model to predict memory accuracy. The analysis predicted memory accuracy (correct or incorrect) on a trial-by-trail basis, from (1) the training condition, (2) the subject-level RSA contrast of similarity between trained and untrained videos in the MPFC, and (3) their interaction. The RSA contrast effect was centered for each counterbalancing group separately.

We next examined general memory reinstatement effects. We compared the pattern similarity between matching video clips during encoding and retrieval (e.g., encode video 1 and retrieve video 1; corresponding to the diagonal of Fig. 3*C*) versus the similarity between mis-matching video clips (e.g., encode video 1 and retrieve video 4; the off-diagonal of Fig. 3*C*). By asking participants to encode and recall the videos in sets of 5, we aimed to ensure that almost all videos could be recalled adequately in the scanner. The post scan recall test verified this was the case in all participants except one (who was excluded from the analysis). Therefore, all encoding and recall trials were entered into the analysis. A posthoc analysis of the reinstatement effects was carried out which excluded trials which could not be later recalled and the results are highly similar to those reported below. The results of this analysis are available from the authors on request.

To compare our results to previous studies (e.g., Bird et al. 2015; Oedekoven et al. 2017), we investigated whether reinstatement effects were modulated by memory accuracy. Focusing only on the encoding-retrieval reinstatement between matching videos, we weighted these values positively and negatively depending on recognition memory accuracy ratings (Fig. 3*D*).

We also investigated whether reinstatement effects were modulated by our training manipulation. Specifically, we examined whether encode-retrieval similarity was higher for the trained clips versus the untrained clips (see Fig. 3*E*).

**Figure 2:**
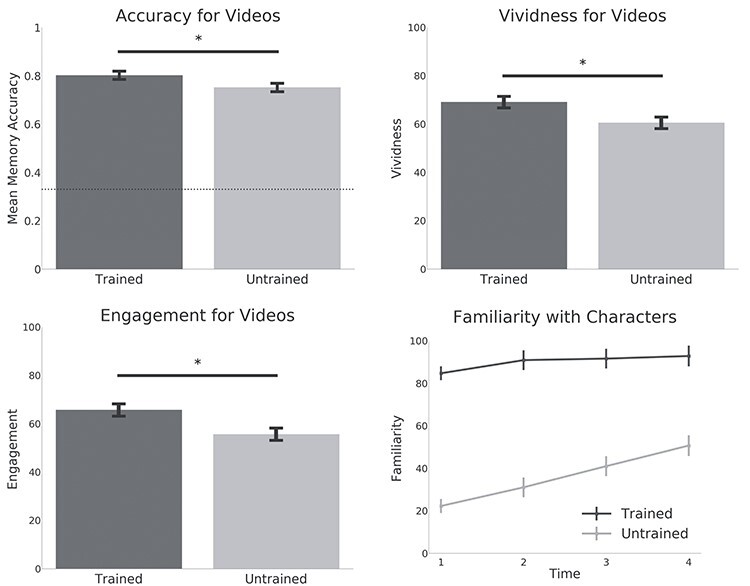
Behavioral results. Bar graphs show memory accuracy for the clips from the trained and untrained shows. Subjective vividness and engagement ratings are also shown. Dashed line indicates chance performance (33%) for the memory test. Stars indicate significance below *P* < 0.01. The line graph shows familiarity ratings for each show (averaged over the characters in each show) after each set of 5 clips. Familiarity ratings after each set were significantly higher for the trained characters when compared with the untrained characters. Graphs represent means and standard errors.

Last, we examined whether training modulated the similarity between recall events. Similarly to the encoding similarity analysis, we examined whether recall trials from the trained show were more similar to each other than recall trials from the untrained show (see Supplementary Fig. 8).

## Results

### Behavioral Results

On the basis of their performance on the free recall test, one participant who failed to recall 11 of the 20 video clips was excluded from the main analysis (see Participants above). For the rest of the participants, performance was good. On average 1.4 video clips were forgotten (range 5–0). Since participants were instructed to give only brief descriptions of the videos, no further analyses of these data were carried out.

As previously reported by Raykov et al. (2020), overall accuracy on the recognition memory task was high (77%, chance level = 33%). Memory was more accurate for the trained clips (80%) versus clips from the untrained show (75%; *t*_26_ = 2.94; *P* = 0.006). Participants also rated remembering the trained clips more vividly (*t*_26_ = 3.38; *P* = 0.002) and found them more engaging (*t*_26_ = 3.71; *P* < 0.001) (see Fig. 2). During an fMRI recall trial, participants could press a button to indicate they had finished recalling the video. On average participants took slightly less time to recall untrained clips (20.8 s) when compared with trained clips (22.13 s; *t*_26_ = 3.3; *P* = 0.002). This is broadly consistent with a beneficial effect of prior knowledge on recall—if we assume that longer recall durations reflect the retrieval of more information. However, we note that we found that recall duration was on average only marginally correlated with accuracy on the recognition test (*t*_26_ = 2; *r* = 0.11; *P* = 0.05).

Familiarity ratings for the characters were averaged separately for each show and each set (4 sets in total). Familiarity ratings showed that although participants felt they knew the characters from the untrained show more after watching the videos, they still did not feel as familiar as the characters from the trained show (see Fig. 2).

**Figure 3:**
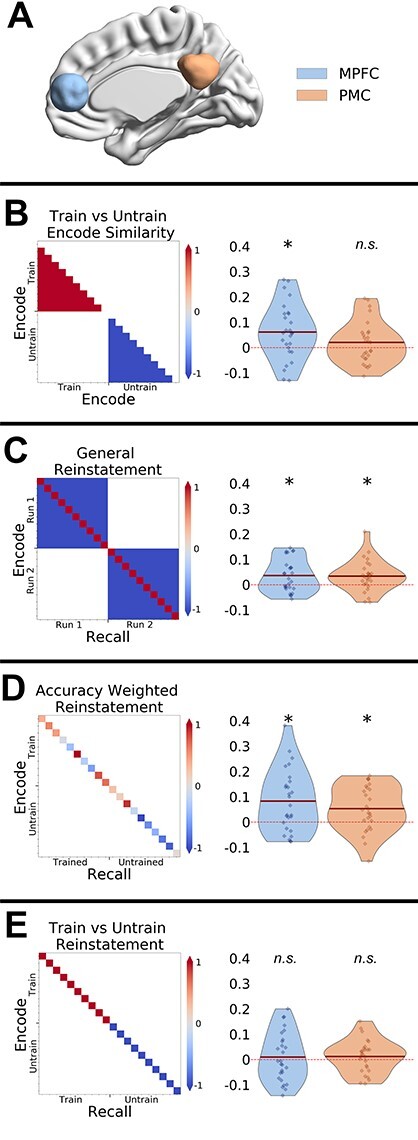
RSA analyses in the MPFC and PMC ROIs. (*A*) ROIs used for the RSA analyses. Rows (*B*–*E*): Prediction matrixes and results are show on separate rows of the figure. Note trained and untrained trials were presented in an interleaved manner and were reordered here for clarity. (*B*) Person knowledge effect on encoding similarity: the contrast identifies whether similarity between clips from the trained show > untrained show. (*C*) Event-specific memory reinstatement effect: contrast identifies whether similarity between matched encoding-recall pairs (same video clip) > nonmatching encoding-recall pairs (different video clips). (*D*) Effect of memory accuracy on reinstatement: contrast identified whether memory reinstatement is greater for better-remembered clips. (*E*) Effect of training on memory reinstatement: contrast identifies whether memory reintatement for the trained show > untrained show. ^*^ = *P* < 0.05, n.s. = nonsignificant.

### Imaging Results

#### Univariate Analyses

The contrast for trained versus untrained videos did not show significant effects in the MPFC ROI [*t*_26_ = 1.0; *P* = 0.16 one-tailed] or the PMC ROI [*t*_26_ = 1.51; *P* = 0.07 one-tailed]. The whole brain contrast also did not show any significant clusters. BOLD activity within the ROIs did not differ as a function of whether or not the event recalled was trained or untrained [MPFC; *t*_26_ = −0.73; *P* = 0.24 one-tailed; PMC; *t*_26_ = −0.39; *P* = 0.34 one-tailed]. The contrast for trained versus untrained recall events also did not show any significant clusters at the whole brain level. In Supplementary Materials we report exploratory analyses examining univariate activity during the onset of the trained and untrained clips (see Supplementary Fig. 2).

### RSA

**Figure 4:**
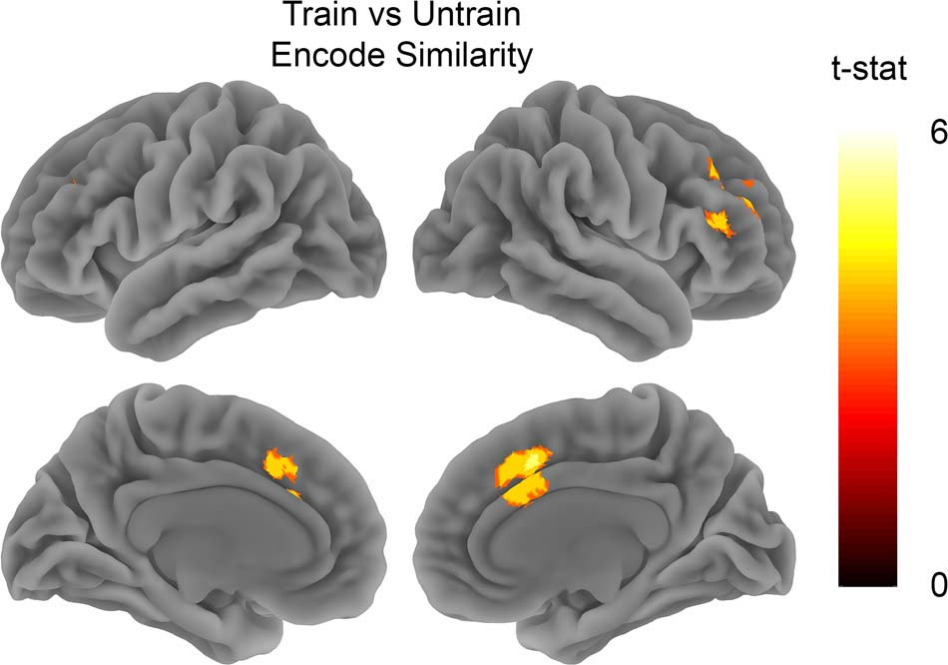
Train versus untrain encode similarity*.* Analysis tested for areas showing higher spatial pattern similarity for clips from the trained show when compared with the untrained show during encoding. Map is thresholded at FWE *P* < 0.05 with voxel threshold of *P* < 0.001.

We compared the similarity between the trained clips and contrasted it with the similarity between untrained clips. We observed similar patterns of activity across all trained clips in the MPFC (*t*_26_ = 2.9, *P* = 0.007), but not in the PMC (*t*_26_ = 1.27, *P* = 0.21) (see Fig. 3*B*). The whole brain analysis also showed that trained clips were more similar to each other during encoding in right IFG, and middle frontal gyrus (see Fig. 4 and Table 1). The searchlight analysis did not reveal any significant clusters that showed the opposite effect of higher similarity between the untrained clips when compared with the trained clips. An exploratory analysis also compared how the similarity between trained clips when compared with the untrained clips changed over time (see Supplementary Figs 3–6). This analysis suggested that the shared pattern of activity between the trained clips in MPFC was maintained throughout the duration of the clips.

**Figure 5:**
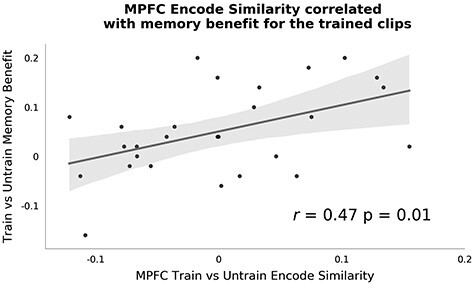
Pattern similarity at encoding correlates with memory benefit. Here, we correlated across individuals the train versus untrain similarity effect in MPFC during encoding with the difference in memory performance between trained and untrained clips. Better memory for the trained clips was correlated with stronger pattern similarity between the trained clips during encoding. Each dot represents data from a single participant.

Interestingly, a posthoc between-subject analysis showed that participants who exhibited higher MPFC similarity between the trained clips compared with the untrained clips also showed a larger boost in their memory performance for the trained clips compared with the untrained clips (see Fig. 5). The correlation between these two variables was significant (*r* = 0.47; *P* = 0.01). To better understand this effect, we fitted a logistic mixed effects model predicting the trial-by-trial memory accuracy from, (1) the training condition, (2) the MPFC trained versus untrained RSA similarity effect, and (3) their interaction. We show the model coefficients in Table 2. The main effect of condition shows that participants were more likely to answer correctly on memory questions from the trained show, which was already shown in Fig. 2. Interestingly, we observed a significant interaction between the training condition and the RSA effect comparing similarity between trained and untrained videos. The MPFC trained versus untrained similarity effect was positively associated with memory accuracy for the trained clips, but not for the untrained clips (see also Supplementary Fig. 9). This suggests that participants that showed higher MPFC trained versus untrained similarity during encoding were also more likely to perform more accurately on the questions from the trained clips.

**Figure 6:**
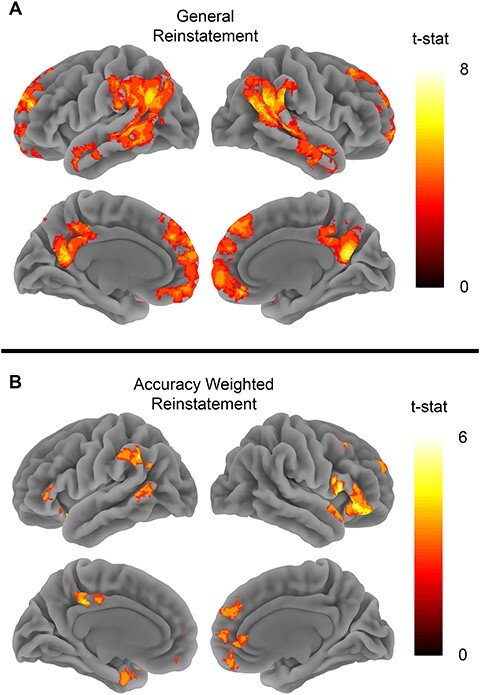
Reinstatement searchlights. (*A*) The general reinstatement analysis tested for areas showing higher spatial pattern similarity between encoding and retrieving the same clip versus different clips. (*B*) Map shows brain areas where reinstatement effects correlated with memory performance. Both map show clusters significant after FWE correction at *P* < 0.05 with voxel defining threshold of *P* < 0.001.

We also observed significant general reinstatement effects in both MPFC (*t*_26_ = 2.9, *P* = 0.007) and PMC (*t*_26_ = 2.89, *P* = 0.007) (see Fig. 3*C*). The whole brain searchlight analysis revealed significant clusters in bilateral angular gyrus, left middle temporal gyrus, left medial frontal gyrus, MPFC, and right middle cingulate (see Fig. 6 and Supplementary Table 1). These results largely replicate previous whole brain findings using similar stimuli (Bird et al. 2015; Oedekoven et al. 2017).

Behavioral accuracy scores were correlated with reinstatement effects in both MPFC (*t*_26_ = 3.3, *P* = 0.002) and PMC (*t*_26_ = 2.32, *P* = 0.02) (see Fig. 3*D*). The whole brain analysis showed that multiple brain areas showed reinstatement effects that were correlated with memory accuracy. Spatial patterns of reinstatement correlated with memory accuracy in bilateral temporal poles, inferior frontal gyrus (IFG), left middle temporal gyrus, precuneus, and ventromedial prefrontal cortex (see Supplementary Fig. 8 and Supplementary Table 3).

**Table 1:** Significant clusters identified for the train versus untrained encode similarity searchlight. Analysis identified areas showing higher spatial pattern similarity during encoding of trained clips when compared with untrained clips. Clusters showing video specific reinstatement effects

Region	*x*	*y*	*z*	Size (voxels)	*T*
Middle cingulate cortex	6	14	46	954	6.12
Middle frontal gyrus	36	32	24	493	4.78

**Table 2:** Betas from a logistic mixed-effect model predicting memory accuracy. The train condition was coded as 1 and the untrain condition was coded as 0. Therefore, the beta for “Train” represents the difference in memory accuracy between conditions. Note MPFC encode similarity is scaled, so that a unit change represents a 0.1 change

	Estimates (log-odds)	SE	*Z*-value	*P*-value
Intercept	1.16	0.11	10.31	*P* < 0.001 ^*^
Train	0.31	0.09	3.29	*P* < 0.001 ^*^
MPFC train vs untrain encode Similarity	-0.08	0.14	-0.55	*P* = 0.57
Train ^*^ MPFC train vs untrain encode similarity	0.33	0.12	2.69	*P* = 0.007 ^*^

Note: ^*^ indicates significant coefficients at *P* < 0.001.

Surprisingly, we did not find any significant clusters at whole-brain level and none of our ROIs (*ps* > 0.34) showed different reinstatement effects for the trained videos when compared with the untrained videos (see Fig. 3*E*). Similarly, we did not observe any differences in the similarity between trained and untrained recall trials (see Supplementary Fig. 8).

## Discussion

In this study, we used the everyday experience of watching episodes of a television sitcom as a way of acquiring new schematic knowledge about two previously unfamiliar people. Participants then watched the characters interacting in novel situations and in unfamiliar locations, while in the MRI scanner. As reported previously, person knowledge resulted in clear-cut improvements in memory for these lifelike events: in comparison with events taken from an untrained sitcom, participants correctly recognized more details about the clips from the trained show and rated them as more vividly remembered and more engaging (see Raykov et al. 2020). Within the MPFC, we found evidence for person-specific representations: fMRI activity patterns were more similar when watching clips involving the trained characters compared with the untrained characters. This encoding similarity effect also predicted better memory performance for the trained clips. Follow-up analyses revealed that this effect was maintained for the duration of the clips, suggesting that the MPFC supports a representation of schematic knowledge about familiar individuals that is active throughout an event. We also replicated recent findings that event-specific patterns of fMRI activity are similar when watching and recalling events, but this effect was not modulated by prior knowledge.

Our fMRI results highlight a role for the MPFC in the activation of person knowledge when processing new events. Patterns of brain activity were more similar within the MPFC when viewing the clips from the trained show compared with the untrained show. Since the trained show was counter-balanced across participants, this effect cannot be due to more basic differences between the two shows, such as the speed of the dialog. Furthermore, since all of the scenes were taken from novel locations not seen in the training episodes, the effects must reflect participants’ differential familiarity with the characters and how they interact. Our results are consistent with studies using different manipulations of prior knowledge in both humans and animals, which have implicated the MPFC in processing of schemas (van Kesteren et al. 2010; Tse et al. 2011; van Kesteren, Beul, et al. 2013; Hsieh and Ranganath 2015; Brod et al. 2016; di Oleggio Castello et al. 2017; Raykov et al. 2020). Lesions to the MPFC have also been suggested to lead to schematic memory deficits (Melo et al. 1999; Ciaramelli et al. 2006; Ghosh et al. 2014; Warren et al. 2014; Spalding et al. 2015). More specifically, the MPFC is often more active when viewing personally known or famous faces compared with unfamiliar faces (Ramon and Gobbini 2018; Wagner et al. 2018). Damage to MPFC has been associated with differential processing of pictures of familiar others (Gilboa et al. 2009; Gilboa and Moscovitch 2017). Here, we extend these studies by identifying a role for the MPFC specifically in the processing of schematic information about people, and by showing that this information is activated during the encoding of extended lifelike events.

Interestingly, in addition to the training-related similarity effect, we observed at the group level, we also found a between-subject correlation, whereby the increase in the similarity between the clips from the trained show in the MPFC was related to the memory advantage for the trained clips. In other words, those individuals who showed the largest knowledge-related increase in similarity during encoding were those who showed the largest boost in memory for the clips from the familiarized show. These findings may shed some light on the mechanisms by which person knowledge boosted recognition memory in our task.

Schematic knowledge has been argued to enable incoming information to be structured and organized (Ericsson and Kintsch 1995). Furthermore, prior knowledge has been suggested to decrease the demands of processing familiar items, freeing up more resources to bind the items to the context in which they are encountered (Reder et al. 2013). Under both of these theories, knowledge serves as a scaffold within which to encode the elements of a novel event. Our finding that activity patterns are more similar across all encoding events involving the trained show, and that the amount of similarity correlates with memory performance, concurs with the notion of a consistent schema that is active during all events from the trained show. By contrast, other theories have proposed that prior knowledge promotes item-specific processing which emphasizes the differences between otherwise similar items, resulting in better encoding of episodic details and making memories more discriminable (Rawson and Van Overschelde 2008; DeWitt et al. 2012). Under such a theory, we might expect to see less pattern similarity during the encoding of the clips from the trained show, but we did not observe this effect (either in our two regions of interest, or elsewhere in the brain).

An alternative explanation for the increased pattern similarity in the MPFC when watching clips from the trained show is that participants are recalling specific memories from the training episodes. The MPFC is associated with episodic memory (see Rugg and Vilberg 2013), and in the present study, we observed memory reactivation effects between encoding and recalling the same events (see also Bird et al. 2015; Chen et al. 2017; Oedekoven et al. 2017; Zadbood et al. 2017). However, we consider this unlikely. This explanation would imply that during all of the different encoding videos, each of which depicts a very different scene, participants were remembering one specific event from a training episode. Moreover, to explain the between-subject correlation with performance, the people whose memory for the clips shown in the scanner was best, would have to be those individuals recalling an unrelated event from a training episode with the most clarity. Instead, it is more likely that they activated a more abstracted representation of the characters, created from their experience of watching them interact in multiple episodes—in other words, a schematic representation (Gilboa and Marlatte 2017). We further note that a recent study of schematic representations of well-learnt event “scripts” highlighted the role of the MPFC, yet it placed very little demand on episodic memory (Baldassano et al. 2018).

Our main analyses involved modeling the whole of the videos as single events. The results therefore suggest that the person schema representations were activated throughout the whole clip. A follow-up analysis of the first 22 s of each video supported this conclusion (see Supplementary Figs 3–6), with the training effect being numerically stronger throughout most of the videos accounting for HRF. This is consistent with the hypothesized role of schemas in providing a set of predictions for how an event might unfold, which can structure and potentially bias information processing towards schema relevant features present in complex events (Dooling and Christiaansen 1977; Thorndyke and Yekovich 1980; Cohen 1981; Baldwin 1992; Ghosh and Gilboa 2014; Franklin et al. 2020). Further support that MPFC may be involved in selecting relevant information comes from a recent study that showed goal-relevant compression of information in MPFC (Mack et al. 2020).

Interestingly, although we observed qualitatively higher similarity among trained clips in PMC this effect did not reach significance. This result contrasts with the results of Baldassano et al. (2018) who found evidence for schematic processing in PMC and parahippocampal cortex. One possible explanation for this discrepancy is the type of schema knowledge used. The airport and restaurant schemas used by Baldassano et al. (2018) are strongly associated with spatial contexts. Both PMC and parahippocampal cortex have been shown to have preference for processing locations and scenes, which might account for the discrepancy between studies (e.g. Marchette et al. 2015; Robin et al. 2018). Furthermore, it should be noted that Baldassano et al. (2018) compared patterns of activity for two well-learned schemas, whereas here we compared pattern similarity between familiar videos versus similarity of unfamiliar videos.

In addition to our analyses in specific ROIs, we also carried out a whole-brain searchlight analysis to investigate the effects of person knowledge on event processing in nonhypothesized regions. This revealed two anatomically close regions which showed the effect of training on encoding similarity: the middle cingulate cortex and the right middle frontal gyrus. Interestingly, the larger effect in the middle cingulate gyrus overlaps with a region recently identified as playing a role in semantic control (Jackson 2020). Semantic control involves the selection and manipulation of information that is appropriate to the current context (Jefferies 2013; Ralph et al. 2017). This accords well with our original suggestion that person knowledge enables us to understand and predict how people will behave in new situations. Further research will clarify whether this region mainly supports processing of knowledge about individual entities (such as people, objects, actions, and words) or whether it also plays a role in broader schematic processing of events, such as by the activation of generic event scripts.

In contrast to the effects of person knowledge during encoding, we did not see any effect on reinstatement or recall. This is surprising since there is ample evidence demonstrating that memory schemas influence the retrieval of information about scenes, events, and stories, particularly when there is a long interval between encoding and test (Bartlett 1932; Brewer and Treyens 1981; Kleider et al. 2008). In these examples, schema-consistent (yet incorrect) information is introduced during memory retrieval, ruling out the possibility that schemas only affect how the information is originally encoded. More recent studies have demonstrated schema-related memory effects after, but not before, a period of memory consolidation (van Kesteren, Rijpkema, et al. 2013; Durrant et al. 2015) and that schemas may only exert their influence on memory retrieval after the fidelity of the original memory has faded (Tompary et al. 2020). Similarly, fMRI studies have found effects of memory schemas on brain activity at retrieval that are apparent after a period of memory consolidation (Wagner et al. 2015; van der Linden et al. 2017). Taken together, these findings suggest that our in-scanner retrieval task may have been too soon after encoding to have revealed fMRI differences during the recall of events from the trained versus untrained show. It is important to note that the memory advantage for the trained versus the untrained show was found using a recognition memory task administered after scanning.

When examining general reinstatement regardless of training, we largely replicated previous work (e.g., Bird et al. 2015; Chen et al. 2017; Oedekoven et al. 2017). We observed memory reinstatement effects in PMC, angular gyrus, middle temporal gyrus, and middle frontal gyrus. Furthermore, reinstatement effects were positively correlated with memory accuracy in many regions of the default mode network.

In summary, our results demonstrate the usefulness of person knowledge in the encoding of new events involving familiar individuals. Furthermore, we identify a role for the MPFC in representing person knowledge and maintaining an active representation of familiar characters throughout the event. This extends recent findings that the MPFC plays a key role in naturalistic event processing by linking prior knowledge with incoming sensory information to interpret events as they unfold.

## Funding

European Research Council (ERC) under the European Union’s Horizon 2020 research and innovation programme (grant agreement No. 819526 to C.M.B.); Economic and Social Research Council studentship (ES/J500173/1 to P.P.R.) and fellowship (ES/V012444/1 to P.P.R.).

## Notes

We thank Ken Norman, Uri Hasson and their labs at Princeton University for training P.P.R. in some of the methods used in this paper and for their helpful advice. Frederick Herbert helped with data collection. *Conflict of Interest*: None declared.

## Supplementary Material

Supplementary_Materials_bhab027

Memory_Questions_bhab027
