# Supplementary material for: Activation of Person Knowledge in Medial Prefrontal Cortex during the Encoding of New Lifelike Events
Source: Cereb Cortex. 2021 Apr 19;31(7):3494–505. doi: 10.1093/cercor/bhab027 (PMC8355471; doi:10.1093/cercor/bhab027)
Supplement: Supplementary_Materials_bhab027 [file supplementary_materials_bhab027.docx]

**Supplementary Materials**


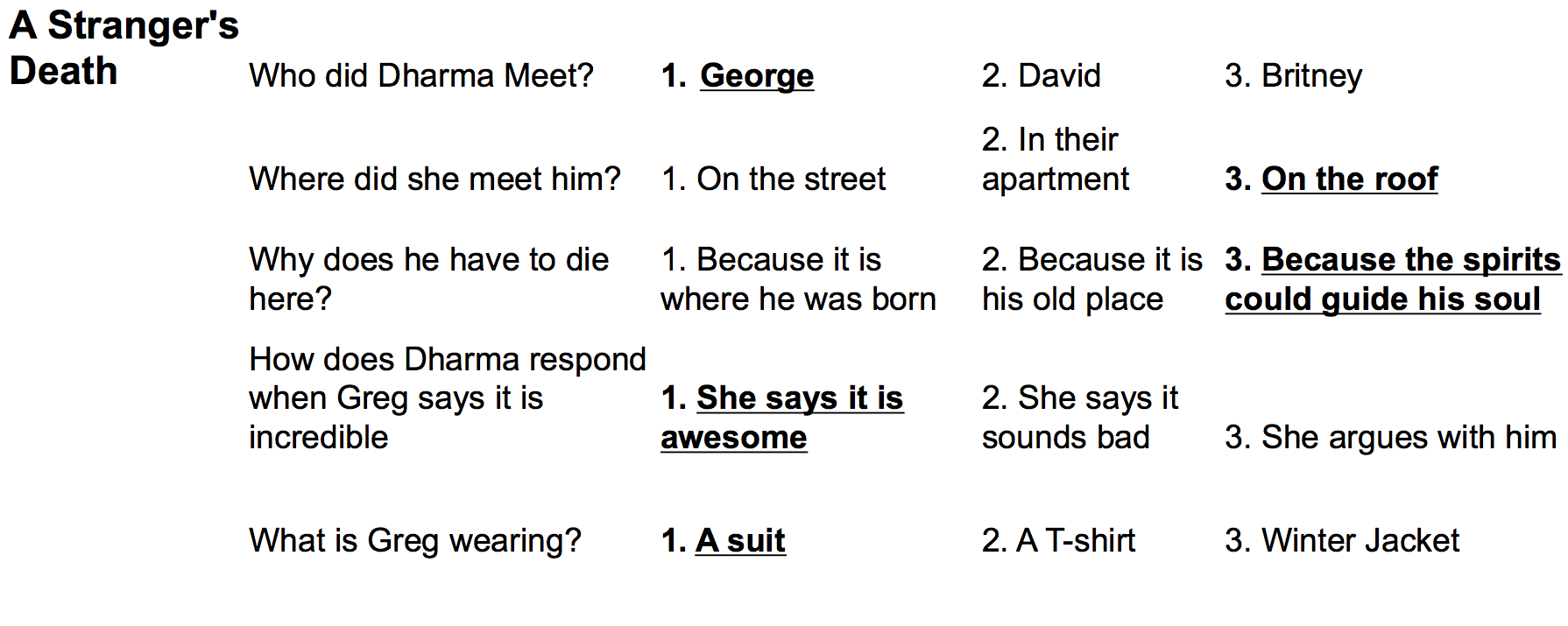


**Supplementary Figure 1** Example questions for a single video clip. The title indicates for which video the questions are. The participants see each question one after the other and have to make a choice between three provided options. The highlighted options are the correct answers (they were not presented to the participants).

**Univariate response at the onset of the encoding and recall trials**

The present study did not report any differences in overall level of brain activity when watching or recalling video clips from the trained, versus the untrained show. However, in a previous study involving the same participants, we reported that both the vmPFC and retrosplenial cortex (RSC) showed higher activity when participants viewed pictures of characters from their trained show versus their untrained show (Raykov et al., 2019). The same participants were shown different pictures of the main characters of the trained and the untrained show. We compared BOLD activity when participants were watching the pictures of characters taken from the trained show to activity to when participants were watching characters from the untrained show. We found higher activity in vmPFC, RSC and the hippocampus when participants were viewing the pictures from the trained show. It is possible that these and other regions may exhibit transitory activity at the beginning of the clips that is not maintained throughout their duration – and that this transient response might be modulated by familiarity with the characters (see Keidel et al., 2018 for a similar effect). We therefore analysed the response to just the onsets of the encoding and retrieval phases. The results are shown in supplementary figure 1. Although there were no effects that were significant at our chosen threshold (p<0.05 FWE corrected for cluster size), some regions were identified at an uncorrected threshold (voxelwise height threshold: p<0.001 uncorrected for multiple comparisons). As can be seen by comparing the results of the picture task with the onsets of the videos task, these effects are broadly similar. During the recall phase there were no regions that showed onset responses that were modulated by training.


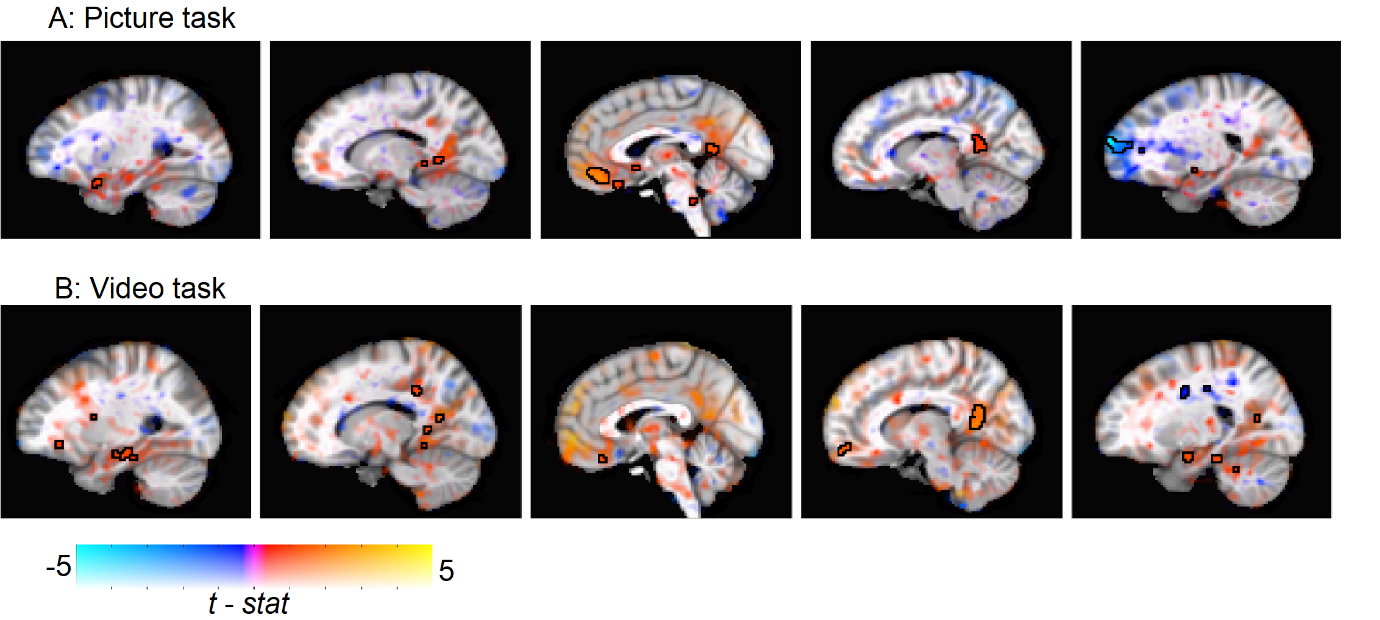


**Supplementary Figure 2** Univariate maps of training effects at the onset of the videos. Brain map shows contrast between trained and untrained pictures (A) and video onsets (B). Warm colours represent higher activations for the trained pictures or videos. Beta values are mapped to colour hue and t-statistics are mapped to transparency. Higher opacity indicates higher t-statistic. Voxels enclosed within black lines are significant at p < 0.001 uncorrected.

**Searchlight Results for Reinstatement Analyses**

**Supplementary Table 1** Significant clusters identified for the General reinstatement RSA contrasting encode-retrieve pattern similarity for matching vs mismatching videos. Clusters showing video specific reinstatement effects.

| Region | x | y | z | Size (voxels) | T |
| --- | --- | --- | --- | --- | --- |
| Right Angular gyrus | 56 | -56 | 32 | 4501 | 8.52 |
| Precuneus | 2 | -58 | 20 | 1647 | 8.22 |
| Left Superior Temporal Gyrus | -48 | -50 | 22 | 5155 | 7.82 |
| Left Superior Frontal Gyrus | -22 | 52 | 20 | 4533 | 7.49 |
| Left Middle Temporal Gyrus | -54 | -4 | -20 | 250 | 5.45 |
| Left Superior Parietal Lobule | -24 | -70 | 54 | 257 | 4.79 |
| Left Superior Medial Gyrus | -4 | 60 | 20 | 4533 | 3.45 |
| Left Middle Temporal Gyrus | -56 | -24 | -8 | 5155 | 3.51 |

**Supplementary Table 2** Significant clusters identified for the Accuracy Weighted Reinstatement RSA - correlating encode-recall reinstatement with memory accuracy. Clusters are significant after FWE correction at p < 0.05 and voxel defining threshold of p < 0.001

| Region | x | y | z | Size (voxels) | T |
| --- | --- | --- | --- | --- | --- |
| Right Inferior Frontal Gyrus | 50 | 18 | 10 | 1250 | 6.91 |
| Right Middle Frontal Gyrus | 42 | 16 | 62 | 502 | 5.7 |
| Frontal Medial Cortex | 2 | 48 | -16 | 377 | 6.2 |
| Left Anterior Temporal Pole | -36 | 10 | -46 | 458 | 5.93 |
| Left Inferior Frontal Gyrus | -36 | 24 | -2 | 402 | 5.9 |
| Left Posterior Cingulate Cortex | -8 | -36 | 36 | 299 | 5.4 |
| Left Middle Temporal Gyrus | -54 | -54 | 8 | 342 | 5.2 |
| Right Inferior Temporal Gyrus | 54 | 2 | -36 | 210 | 5.3 |
| Left Middle Temporal Gyrus | -56 | -66 | -4 | 257 | 5.10 |
| Left Middle Orbital Gyrus | 0 | 56 | 4 | 255 | 5.1 |
| Right Superior Frontal Gyrus | 18 | 58 | 32 | 561 | 4.94 |
| Left Supramarginal Gyrus | -56 | -48 | 34 | 206 | 4.86 |
| Right Superior Medial Gyrus | 10 | 20 | 50 | 187 | 4.1 |
| Right Parahippocampal Cortex | 16 | 0 | -20 | 177 | 4.63 |
| Right Middle Frontal Gyrus | 38 | 56 | 20 | 169 | 3.82 |

**Timepoint-by-timepoint RSA**

We observed similar patterns of activity between the clips that participants were trained on, compared to clips from the untrained show. To better understand the time-course of this effect we conducted the same RSA analysis separately for each TR in both MPFC and PMC. We also show the same contrast in a occipital region in which we did not expect to observe training-related differences. The occipital ROI was defined from the Harvard-Oxford cortical probability atlas as the voxels that were with at least 50% probability belonging to the occipital pole. We tested whether patterns of activity for the trained clips are more similar to each other than compared to the untrained clips as was performed for the Trained vs Untrained Encode similarity task. The main differences are that here we did that without estimating a single pattern for each clip. We initially regressed out signal from the white matter, CSF, and motion parameters from each run. We then applied a 6mm FWHM gaussian smoothing kernel. For each clip we selected its first 28 TRs. We chose 28 TRs since that was the duration of the shortest clip (22 sec). We ran the same RSA analyses comparing similarity between trained and untrained clips independently for each of the 28 TRs and plotted below the resulting contrast. This meant that, the correlation matrix for the 1^st^ RSA analyses used the 1^st^ TR from each of the 20 clips (see Supplementary Fig. 2). Note video presentation was TR-locked so 1^st^ TR signalled the onset of the clip. The below plots support the argument that participants maintained a schematic representation throughout the video duration (see Supplementary Fig. 3-5). However, we note that our design was not optimised to test for this directly, due to differences in clip duration (22sec – 45 sec).


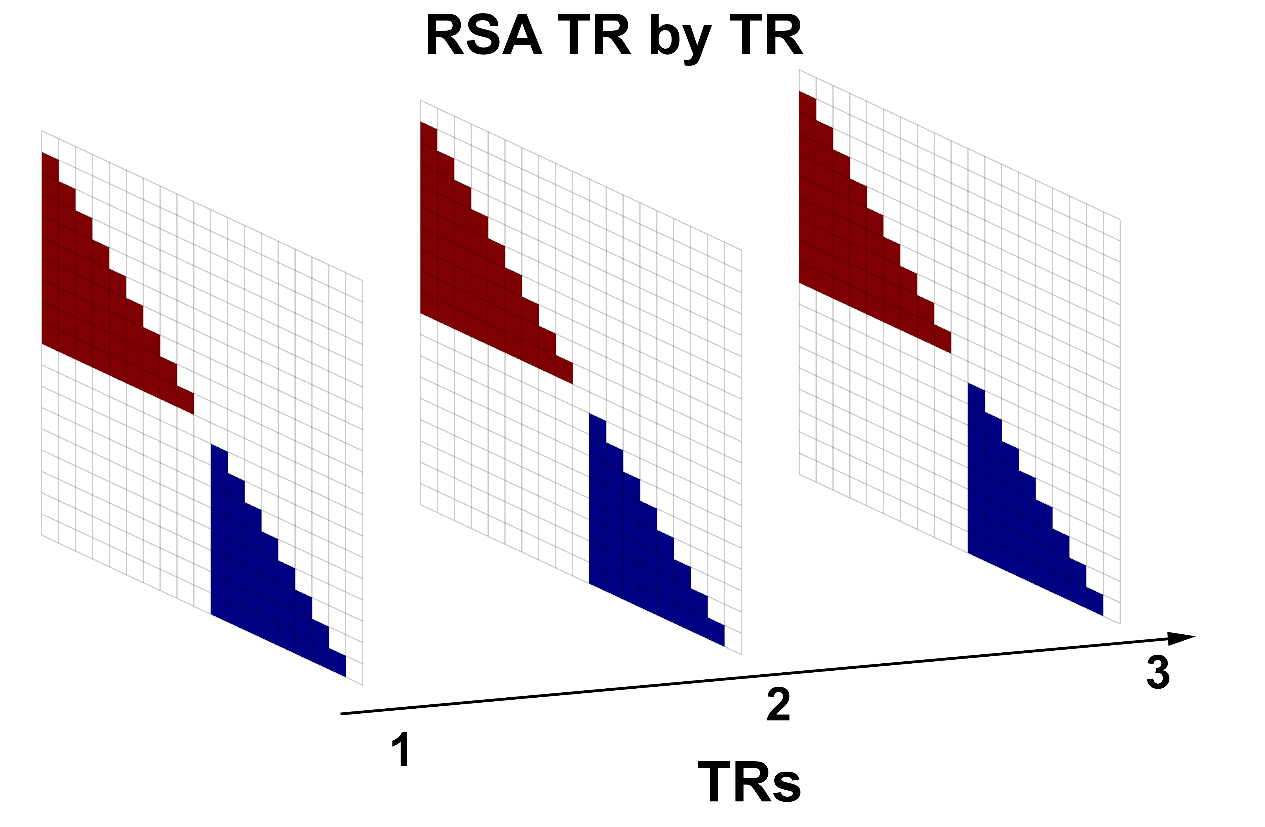


**Supplementary Figure 3** Schema of Temporal RSA. The figure illustrates the TR by TR RSA analysis. We repeated independently across the first 28 TRs of all videos the Trained vs Untrained Encode similarity analysis reported above. For instance, for the first TR RSA analyses to construct the correlation matrix we used the 1^st^ TR for each of the videos and correlated them. We used only the first 28 TRs, because this was the shortest clip.


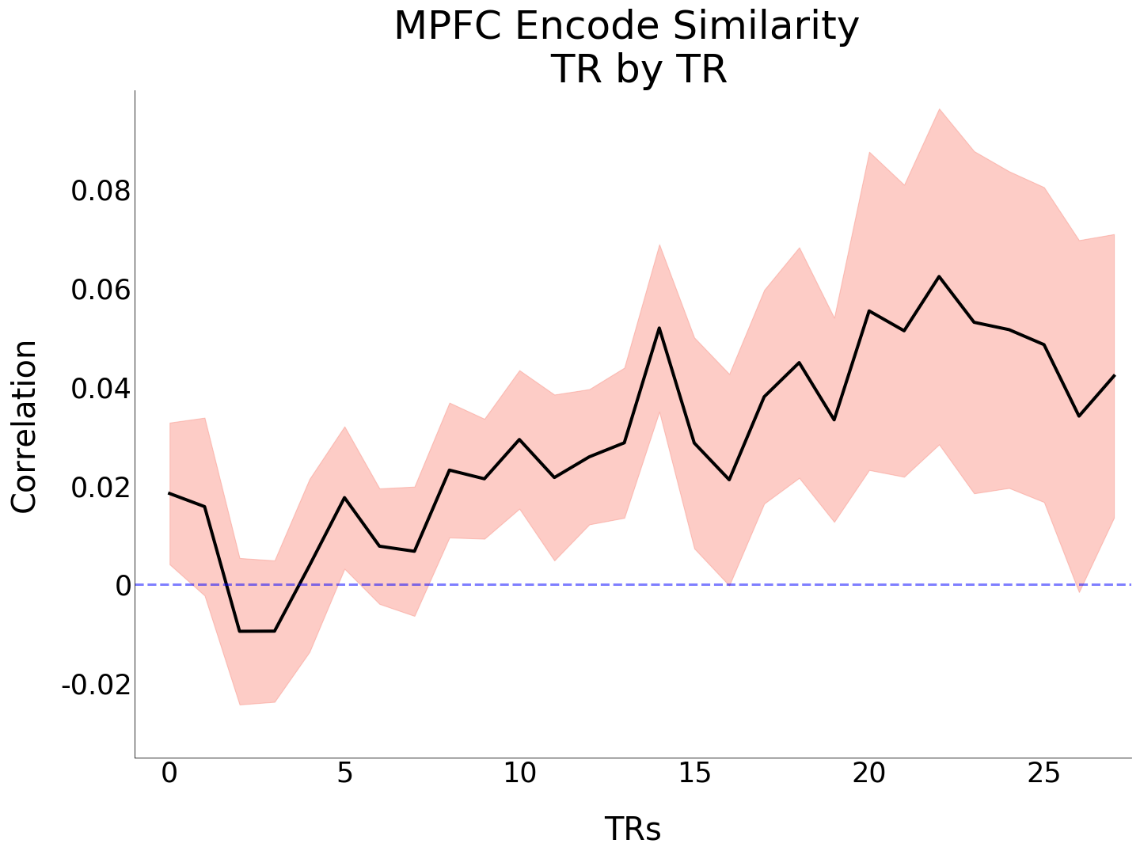


**Supplementary Figure 4** Results for MPFC TR-TR RSA. This shows the contrast correlation in MPFC comparing the Trained vs Untrained video similarity. The mean difference across subjects and standard error is plotted for the first 28 TRs of all clips.


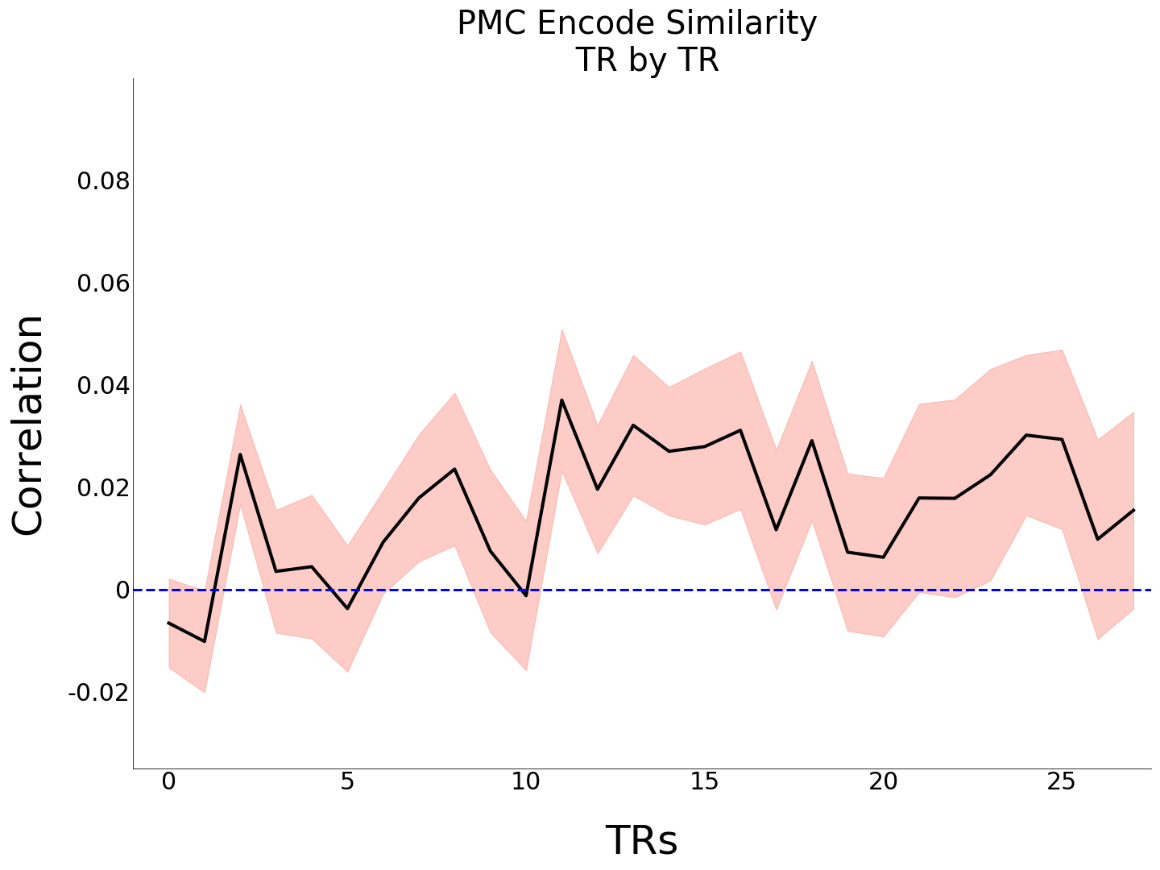


**Supplementary Figure 5** Results for PMC TR-TR RSA. This shows the contrast correlation in PMC comparing the Trained vs Untrained video similarity. The mean difference across subjects and standard error is plotted for the first 28 TRs of all clips.


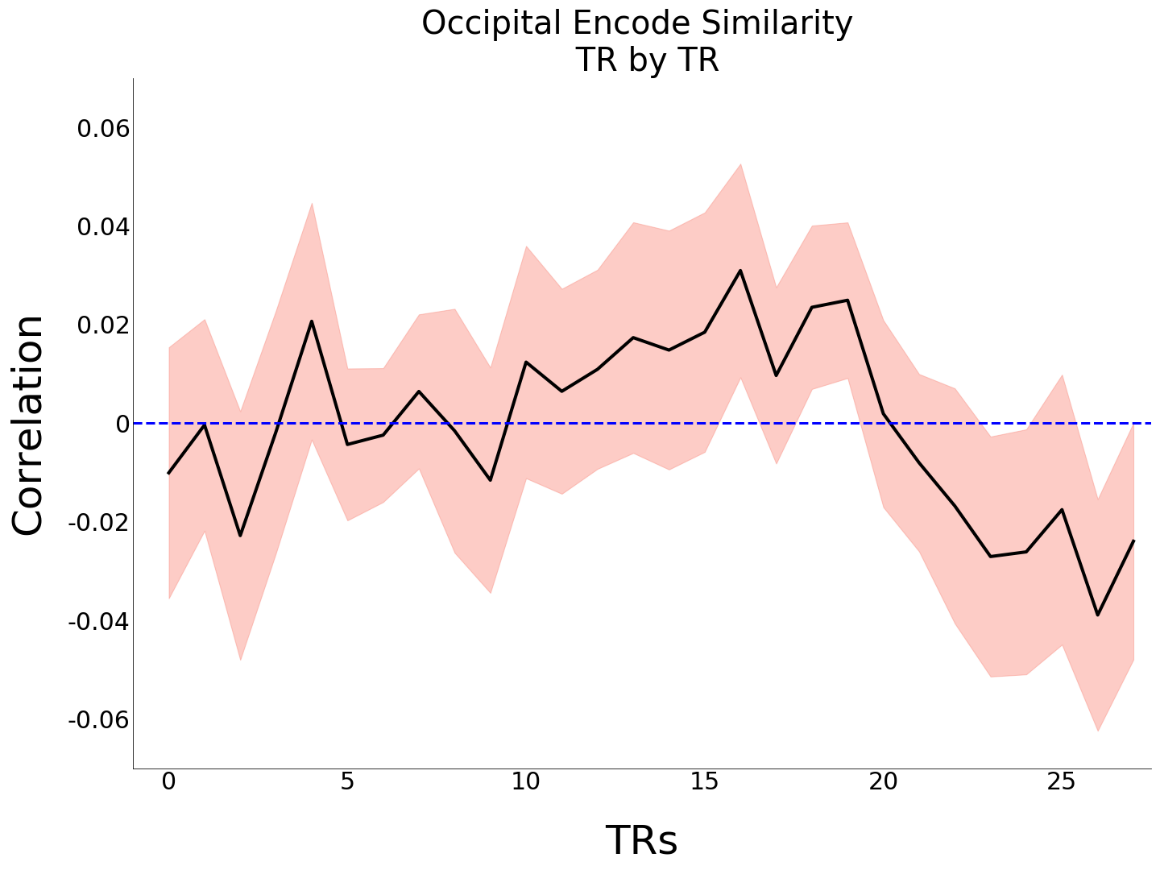


**Supplementary Figure 6** Results for occipital ROI TR-TR RSA. This shows the contrast correlation in occipital ROI comparing the Trained vs Untrained video similarity. The mean difference across subjects and standard error is plotted for the first 28 TRs of all clips.

**Results from different MPFC ROI**

To verify that our results were not due to the selection of a relatively small MPFC ROI, we show the effects in the larger MPFC ROI from Shirer et al. (2012) for the Train vs Untrain Encode similarity contrast. We tested whether similarity of training videos was higher than untrained videos in larger MPFC ROI, and show that the results are consistent across ROIs.

*
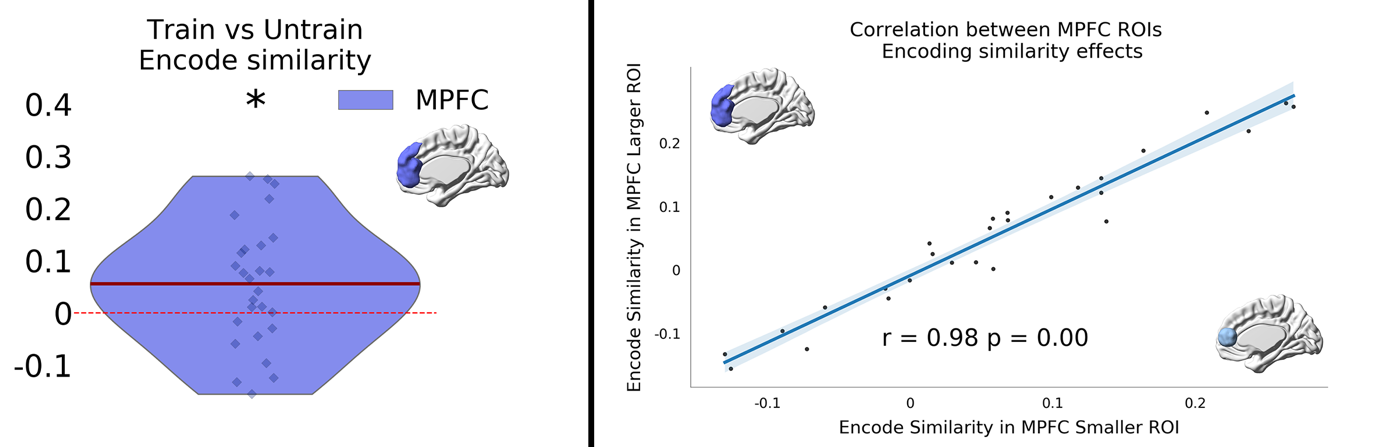
*

**Supplementary Figure 7.** Train vs Untrain similarity MPFC ROI. On the left we show higher similarity for the trained vs untrained clips during encoding in a larger MPFC ROI. On the right we show the correlation between results from this contrast in the larger MPFC and the smaller, spherical MPFC used in main analyses.

**Train vs Untrain Recall Similarity**

We carried out an additional analysis to compare whether trained clips are more similar to each other versus untrained clips during recall. This analysis is identical to the encode similarity reported in the main text (Fig. 4-A), except that it was performed on the recall data.

We did not observe any shared patterns of activity between the trained clips during recall, in either the MPFC (t_26_ = 0.3; p = 0.76) or the PMC (t_26_ = 0.18; p = 0.85). This is in line with the lack of observed modulation of reinstatement by training. In the main text we have speculated on possible explanations for these results.


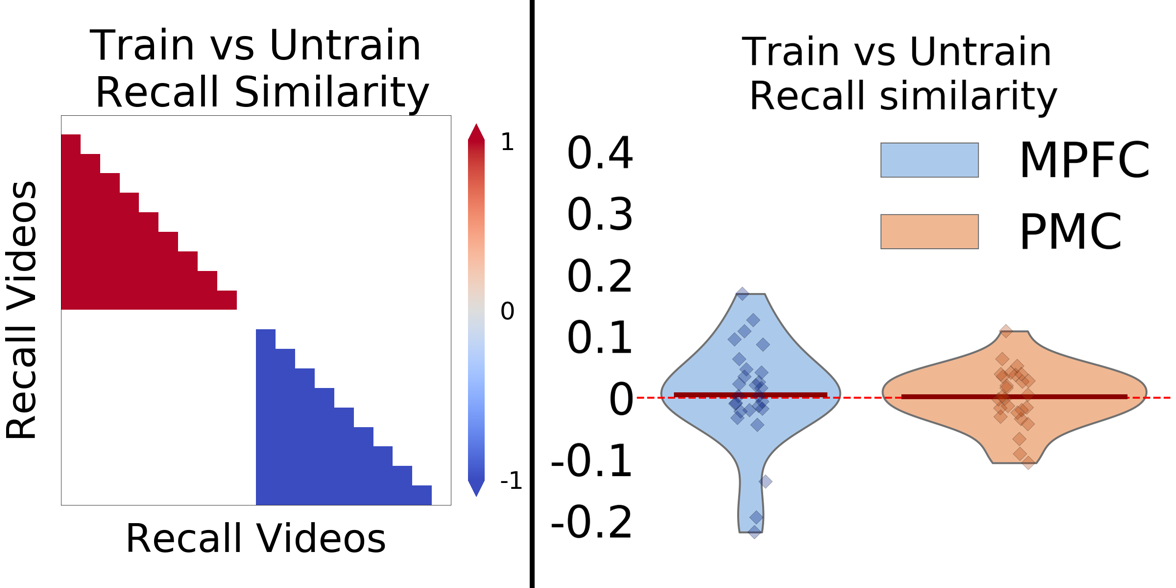


**Supplementary Figure 8.** Here we report the trained vs untrained similarity between recall trials. We examined whether patterns of activity were more similar among trained recall trials when compared to untrained recall trials. On the left the contrast matrix for the analysis is shown. On the right the results in MPFC and PMC are shown. We did not observe any significant difference between trained and untrained recall trials.

**Memory predicted from MPFC Encode Similarity**


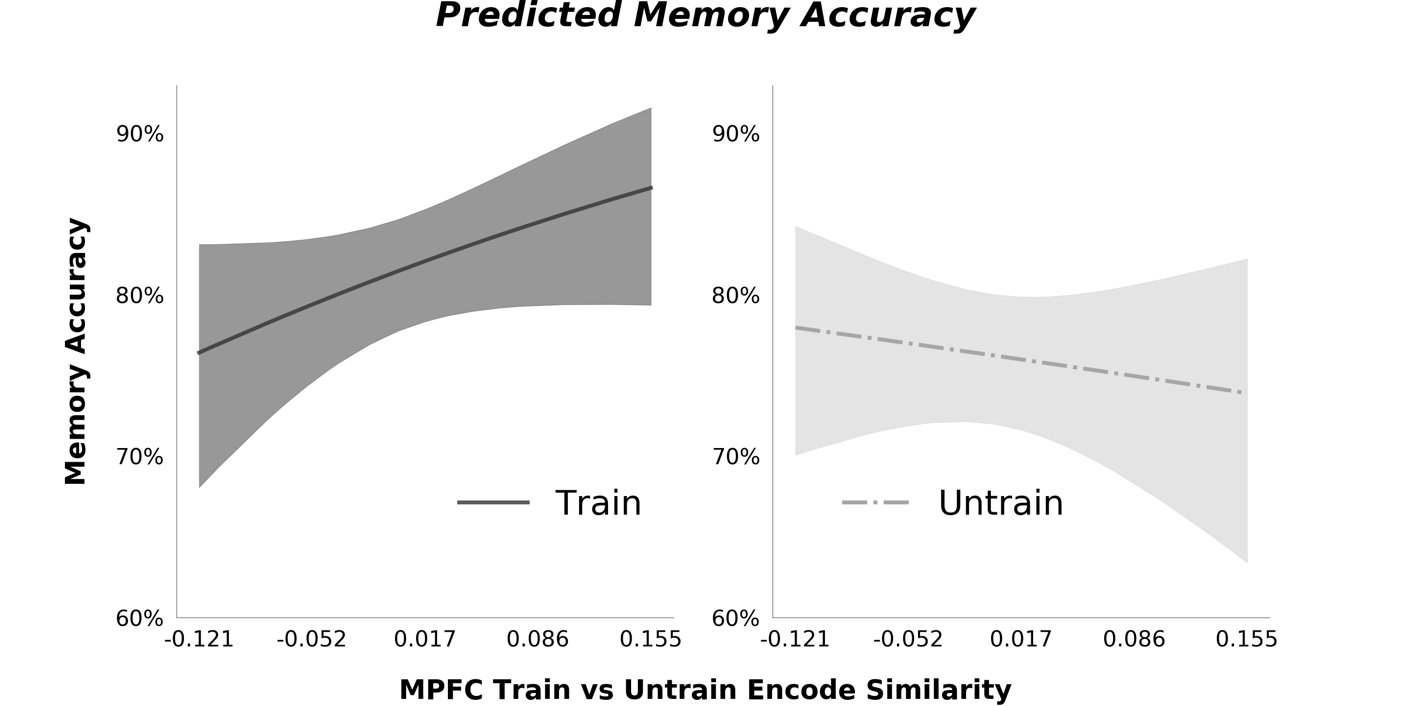


**Supplementary Figure 9** Results from logistic mixed-effect model. Predicted Probabilities of memory accuracy. The plot shows the relationship between the subject-level MPFC Train vs Untrain Encode Similarity effect memory accuracy in both the trained and untrained conditions.

**List of Training Episodes**

**Show:** Mad About You – main character portrayed by actors Paul Reiser and Helen Hunt (see <https://en.wikipedia.org/wiki/List_of_Mad_About_You_episodes> ):

Season 1 Episode 1: *Romantic Improvisations* – Paul and Jamie try to find a moment to themselves while dealing with an unplanned dinner party.

Season 2 Episode 2: *Murray’s Tale* – Jamie’s sister takes Paul’s dog (Murray) for a walk and returns the wrong dog. Paul and Jamie start looking for Murray.

Season 2 Episode 3: *Bedfellows* – Paul’s father has a heart attack and Paul and Jamie visit him in the hospital, and later visit Paul’s mother.

Season 2 Episode 5: *So I Married a Hair Murder* – Jamie is bored as she is recently unemployed and she is looking for new things to do. She ruins Paul’s hair.

Season 2 Episode 10: *It’s a Wrap* – Jamie is jealous that Paul likes his job, and decides to go back to college. Paul is having issues with his boss.

Season 3 Episode 21: *Cake Fear –* A set of flashbacks show Paul and Jamie’s at multiple birthday parties throughout the years.

**Show:** Dharma and Greg – main characters portrayed by actors Thomas Gibson and Jenna Elfman (see <https://en.wikipedia.org/wiki/List_of_Dharma_%26_Greg_episodes> )

Season 2 Episode 8: *Like, Dharma’s Totally Got a Date* – Dharma goes to a high school prom with a kid who asked her out.

Season 2 Episode 24: *The* *Dating Game –* Dharma and Greg decide to pretend to go on a first date. Dharma gets offended and Greg has to beg her to forgive him.

Season 3 Episode 9: *Law and Disorder –* Greg practices law from his home, however Dharma constantly interferes.

Season 3 Episode 12: *Looking for the Goodbars –* Dharma and Greg are searching for new friends.

Season 3 Episode 16: *Weekend at Larry’s –* Dharma and Greg spent time in Larry’s house and dig up a skeleton in the garden.

Season 4 Episode 8: *Charma loves Greb –* Dharma’s plans for Greg’s birthday go wrong.

**List of clips used in scanner**

**Show** Mad About You

Season 1 Episode 2, (duration 29s): *Smell my eye* – Paul is sprayed in the eye with a cologne.

Season 2 Episode 7, (duration 39s): *Stuck in a Dinosaur* – Paul and Jamie are in a museum and Paul gets stuck in the mouth of a fossil dinosaur

Season 3 Episode 10, (duration 21s): *Stepped in Something –* Paul and Jamie are outside an apartment building and Paul steps in veal.

Season 4 Episode 6, (duration 37s): *Birthdays* – Paul and Jamie discuss their birthdays, before meeting Yoko.

Season 1 Episode 13 (duration 37s): *Code Word* – Paul and Jamie are about to meet a client and Paul wants to make up a code word in case things go wrong.

Season 2 Episode 18 (duration 34s): *Recognition –* Paul and Jamie have lost a tape of them, that has ended up in a stranger’s possession. They are worried he will recognize them.

Season 3 Episode 13 (duration 29s): *Getting Mail Out* – Paul and Jamie try to get a letter out of a mailbox because of a typo Jamie has made.

Season 4 Episode 13 (duration 33s): *Jail* – Paul and Jamie are in jail. Paul used his single phone call to check his voice mail

Season 1 Episode 11 (duration 43s): *First date* – Paul and Jamie meet for the first time at a Christmas party.

Season 3 Episode 18 (duration 25s): *Goodbyes* – Paul and Jamie are in the subway to the airport and say their goodbyes now, because they are in a hurry.

**Show** Dharma and Greg

Season 1 Episode 7 (duration 27s): *A stranger’s Death* – Dharma has invited a stranger to die peacefully in their apartment.

Season 1 Episode 8 (duration 41s): *Car Embarrassment* – Greg is worried Dharma will embarrass him in front many people.

Season 2 Episode 10 (duration 32s): *Bought store* – Dharma spontaneously buys a store without knowing what she will sell

Season 5 Episode 16 (duration 28s): *Office Gift –* Dharma gives Greg a picture of herself for their anniversary.

Season 3 Episode 22 (duration 29s): *Man Cave* – Dharma finds Greg has a secret storage where he keeps his old stuff and gets offended.

Season 4 Episode 19 (duration 22): *Performance* – Dharma and Greg are about to see a performance; they discuss the last play they saw.

Season 5 Episode 9 (duration 45s): *Getting Married* – Dharma and Greg are at an airport, in a hurry for a flight, but Dharma is helping someone get married.

Season 5 Episode 17 (duration 41s): *Corporate Wife* – Dharma goes to Greg’s office party even though she does not fit well with his colleagues.

Season 5 Episode 20 (duration 41s): *Aliens –* Dharma and Greg discuss the plausibility of existence of aliens.

Season 5 Episode 19 (duration 28s): *Book Store* – Dharma and Greg buy adult books.
